# Energy Development Linked with Earthquakes

**DOI:** 10.1289/ehp.120-a388

**Published:** 2012-10-01

**Authors:** Bob Weinhold

**Affiliations:** Bob Weinhold, MA, has covered environmental health issues for numerous outlets since 1996. He is a member of the Society of Environmental Journalists.

At least 13 U.S. states^1^ and 20 additional countries^2^ have experienced earthquakes in recent decades associated with subsurface energy development, according to a report released 15 June 2012 by the National Research Council (NRC).^3^ Human-induced earthquakes—which sometimes have surprised residents in areas with little history of seismic activity—have usually been relatively mild, although occasionally entering the range of magnitude 5.0–7.3.^3^ These earthquakes have not led directly to any reported deaths or severe structural devastation (although unreported deaths, injuries, and damage are possible), and have numbered relatively few given the extent of energy-related drilling, injection, and associated operations, according to the authors. But the recent global escalation in subsurface operations spanning the full cycle of energy production—including oil and gas extraction, geothermal energy projects, and carbon capture and storage (CCS)—suggests the number of earthquakes linked to these processes could plausibly increase in tandem.

The primary cause of earthquakes related to energy development, the authors concluded, is when the extraction or injection of fluids changes the balance of stresses around an existing fault (which may not have been previously identified). This can cause the fault to slip or move, generating an earthquake.^3^ With this in mind, the authors predict the type of energy-related activity most prone to inducing earthquakes is large-scale CCS, since it involves continual, long-term, high-volume pressurized injection of liquefied carbon dioxide without concurrent removal of substantial quantities of other fluids. However, they point out that no large-scale CCS projects have been built yet, only small pilot projects, so the risk remains uncertain.

Injection of waste fluids derived from oil or gas operations, including hydraulic fracturing (fracking) projects, is another process of concern, because such procedures introduce large volumes of fluids underground over short time periods without compensatory extraction of similar volumes.^3^ At the April 2012 annual meeting of the Seismological Society of America, investigators from the U.S. Geological Survey (USGS) described a sharp rise over the past decade in earthquakes that were potentially related to waste injection throughout the United States. The authors say the number of earthquakes greater than magnitude 3.0 in the central United States rose from about 21 per year in 1970–2000 to 29 per year in 2001–2008, then to 50, 87, and 134 in 2009, 2010, and 2011, respectively. The findings are expected to be published later this fall.^4^

Earthquakes strong enough for the public to feel—so-called felt earthquakes—have been linked with this kind of injection in a number of locations, and many more largely imperceptible events may also be occurring. A study conducted in Texas found that a grid of temporary seismographs installed near injection wells detected numerous low-magnitude earthquakes that went unreported by the USGS National Earthquake Information Center.^5^

Fracking itself seems to directly create very few felt earthquakes (with at least two documented exceptions: a pair of quakes near a Blackpool, England, drilling site in the spring of 2011,^3^ and a string of events near multiple drilling sites in northeastern British Columbia from 2009 through 2011^6^). Geothermal energy development—which generally involves extraction of steam or hot water from subsurface geological formations, sometimes enhanced by fracking and/or injection of liquids with or without pressure—has triggered felt earthquakes in numerous settings and sometimes in high numbers. Geothermal-related seismic activity is thought to be tied to both fluid imbalances and temperature changes.^3^

The committee says two practices might help prevent induced earthquakes: 1) working more diligently to maintain a fluid balance and 2) doing better site-specific subsurface investigations prior to beginning energy development. With current practices, a detailed site-specific subsurface investigation for waste injection is seldom undertaken, the authors say.

Even if such investigations were to occur, there currently are no predictive models that can interpret the acquired data in a useful fashion.^3^ “[We] did not make an analysis of the time it would take to develop accurate predictive models,” says Elizabeth Eide, director of both the NRC study and the National Academy of Sciences Board on Earth Sciences and Resources. “The speed with which research could proceed would depend in part on availability of data with which to test the models, funding to support the research, and other factors.”

In the interim, the committee recommends that federal and state agencies adopt ways to coordinate more closely to address related issues, and that energy companies take a more active role in preventing potential earthquakes. The report authors also note it’s impossible at this point to accurately predict the location, timing, or magnitude of such events. They therefore recommend that regulatory agencies should collect data on fluid injection and also consider requiring collection and analysis of data to identify faults for hazard and risk analysis before energy development projects commence in areas with high-density structures and populations.^3^ The committee wasn’t asked to recommend specific areas where such conditions apply, Eide says.

The U.S. Environmental Protection Agency (EPA) has some regulatory authority over fluid injection under the Safe Drinking Water Act and is conducting a lengthy investigation of fracking and its potential impacts on drinking water.^7^ However, that project will not address earthquakes, nor could an EPA spokeswoman identify any efforts under way to assess, and possibly regulate, seismic risks tied to energy development.^8^ However, this agency and others are working through the Underground Injection Control National Technical Workgroup to develop information that government agencies and industries might use to understand and mitigate risks from underground injection of petroleum drilling waste. A final report was originally expected in 2011;^9^ a revised release date has not been announced.

The U.S. Department of the Interior includes the Bureau of Land Management, another federal agency with significant regulatory control over energy development. An Interior spokesman declined to comment on whether the agency would defer energy development in vulnerable areas until accurate predictive models are available.
